# Relationship between the Number of Deaths Due to Renal Failure and Air Temperature Parameters in Hokkaido and Okinawa Prefectures, Japan

**DOI:** 10.3390/epidemiologia2010006

**Published:** 2021-02-04

**Authors:** Yoshiro Mori, Hiromi Suzuki, Nobuyuki Miyatake, Masaki Bando, Hiroshi Kinoshita, Naoko Tanaka, Setsuo Okada

**Affiliations:** 1 Department of Hygiene, Faculty of Medicine, Kagawa University, Miki, Kagawa 761-0793, Japan; tanzuki@med.kagawa-u.ac.jp (H.S.); miyarin@med.kagawa-u.ac.jp (N.M.); banmasa0802@gmail.com (M.B.); 2 Sakaide City Hospital, Sakaide, Kagawa 762-8550, Japan; hosp02@city.sakaide.lg.jp; 3 Department of Forensic Medicine, Faculty of Medicine, Kagawa University, Miki, Kagawa 761-0793, Japan; kinochin@med.kagawa-u.ac.jp (H.K.); ntanaka@med.kagawa-u.ac.jp (N.T.)

**Keywords:** Hokkaido, Okinawa, renal failure, air temperature, ecological study

## Abstract

*Background*: The aim of the present study was to investigate the relationship between the number of deaths due to renal failure and air temperature parameters in Hokkaido (the northernmost region) and Okinawa (the southernmost region) prefectures, Japan. *Methods*: Monthly data on the number of deaths due to renal failure between January 2008 and December 2016 and annual population data were collected from the Hokkaido and Okinawa official prefecture websites. Air temperature parameters were obtained from the Japan Meteorological Agency. The relationship between the number of deaths due to renal failure and air temperature parameters was evaluated by an ecological study. *Results*: The number of deaths due to renal failure (per 100,000 people/month) in Hokkaido and Okinawa were 2.28 ± 0.30 and 1.17 ± 0.31. In Hokkaido, the number of deaths due to renal failure negatively correlated with air temperature parameters in both sexes. The number of deaths due to renal failure was significantly higher in January than from June to September in all subjects. However, in Okinawa, no significant difference was observed among months. *Conclusions*: The present results suggest that the relationship between the number of deaths due to renal failure and air temperature parameters differs between Hokkaido and Okinawa.

## 1. Introduction

The number of deaths in Japan has been increasing [1], and the number of deaths due to renal failure per 100,000 people/month) in 2018 was 26,080 (21%), which ranked as the eighth leading cause of death in Japan [2]. More than 300,000 patients are currently receiving chronic hemodialysis, and the Japanese Society for Dialysis Therapy expects this number to increase [3]. Therefore, strategies to prevent and reduce the number of deaths due to renal failure are urgently required in Japan.

Many factors have been reported to affect renal failure [4,5,6,7]. Environmental factors, such as air temperature parameters, have been associated with the number of deaths due to renal failure [8,9,10,11,12]. 

Consistent with this finding, we previously reported that the number of deaths due to renal failure was closely associated with air temperature parameters and was significantly higher in January than in other months in Gifu prefecture, which is centrally located in Japan [13]. However, the relationship between the number of deaths due to renal failure and air temperature parameters in other areas and throughout Japan remains unclear.

Therefore, we herein investigated the relationship between the number of deaths due to renal failure and air temperature parameters in Hokkaido (the northernmost) and Okinawa (the southernmost) prefectures, Japan using an ecological study. 

## 2. Methods

### 2.1. Study Area

**Figure 1:**
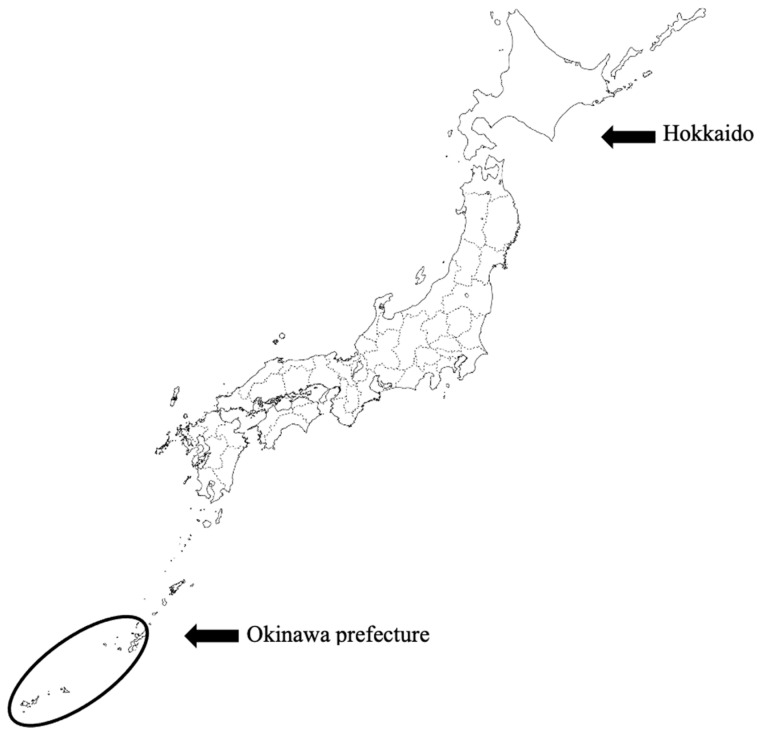
Hokkaido and Okinawa prefectures, Japan.

Hokkaido is located in the northernmost region of Japan (latitude 43°03′51″ north (N)) [14]. Its population and area are 5,382,000 and 83,424 km^2^, while its population density is 68.6 people/km^2^ [15]. Okinawa prefecture is located in the southernmost region of Japan (latitude 26°12′45″ N) [14]. Its population, area, and population density are 1,434,000, 2281 km^2^, and 628.4 people/km^2^ [15], respectively (Figure 1).

### 2.2. Deaths Due to Renal Failure

Monthly data on the number of deaths due to renal failure (between January 2008 and December 2016) in Hokkaido and Okinawa, Japan, were collected from each official prefecture website [16,17]. Annual population data (January) on each prefecture were also obtained from official websites [18]. The monthly population-adjusted number of deaths due to renal failure was then calculated (per 100,000 people/month).

### 2.3. Air Temperature Parameters

We collected information on the following monthly air temperature parameters (same observation period): mean air temperature (°C), mean of the highest air temperature (°C), mean of the lowest air temperature (°C), the highest air temperature (°C), and the lowest air temperature (°C), from the Japan Meteorological Agency [19].

### 2.4. Statistical Analysis

Data were expressed as the mean ± standard deviation (SD). The relationship between the number of deaths due to renal failure and air temperature parameters was evaluated by a simple correlation analysis. Comparisons of the number of deaths due to renal failure among months were performed using the Kruskal–Wallis test and Steel test, with *p* < 0.05 indicating a significant difference. JMP Pro 15 (SAS Institute Inc., Cary, NC, USA) was used for this analysis.

### 2.5. Ethics

All data used in the present study were obtained from official websites. The Ethical Committee in Sakaide City Hospital, Sakaide, Japan approved this study (Number: 2020-011, Date: 30 October 2020).

## 3. Results

Table 1 shows clinical data on the number of deaths due to renal failure and air temperature parameters in Hokkaido and Okinawa, Japan. The numbers of deaths due to renal failure (per 100,000 people/month) in Hokkaido and Okinawa were 2.28 ± 0.30 and 1.17 ± 0.31, respectively. The number of deaths due to renal failure in all subjects was significantly higher in Hokkaido than in Okinawa. Mean air temperatures in Hokkaido and Okinawa were 9.47 ± 9.27 and 23.31 ± 4.49 °C, respectively. All air temperature parameters were significantly lower in Hokkaido than in Okinawa (Table 1).

We investigated the relationship between the number of deaths due to renal failure and air temperature parameters (Table 2). In Hokkaido, the number of deaths due to renal failure negatively correlated with all air temperature parameters in all subjects, men, and women. In Okinawa, the number of deaths due to renal failure also negatively correlated with air temperature parameters, except for the lowest air temperature, in all subjects and men. No correlation was observed between the number of deaths due to renal failure and air temperature parameters in women.

**Figure 2:**
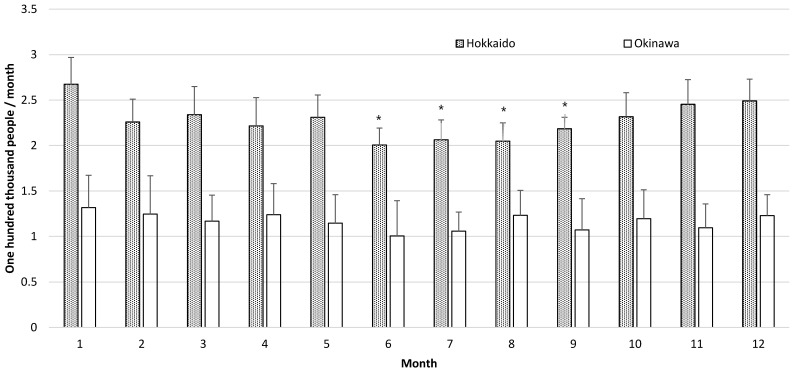
Comparison of the number of deaths due to renal failure classified by month in Hokkaido and Okinawa, Japan. * *p* < 0.05 vs. January.

We then compared the number of deaths due to renal failure by month in Hokkaido and Okinawa (Table 3). In Hokkaido, the number of deaths due to renal failure in January was the highest among months, and it was significantly higher than that from June to September in all subjects (Figure 2). The number of deaths due to renal failure was significantly higher in January than in June and September in men and from June to August in women. However, in Okinawa, no significant differences were observed in the number of deaths due to renal failure among months in all subjects, men, and women.

## 4. Discussion

In the present study, we investigated the relationship between the number of deaths due to renal failure and air temperature parameters. In Hokkaido, the number of deaths due to renal failure was closely associated with air temperature parameters. However, in Okinawa, no significant differences were noted in the number of deaths due to renal failure among months in this ecological study.

Previous studies examined the relationships among renal failure, season, and air temperature parameters. Phillips et al. reported the highest proportion of acute renal failure episodes from January to March (26.2%) and the lowest from October to December [8]. Obi et al. examined seasonal variations in transition, mortality, and kidney transplantation among patients with end-stage renal disease in the United States of America (USA), and they found that the rate of transition was the highest in January [9]. In Japan, the number of acute kidney injuries was the highest in January among hospitalized patients [10]. Iseki et al. reported a strong correlation between the transition for hemodialysis and ambient air temperature in patients with chronic renal failure in Okinawa, Japan [11]. Goto et al. also found that the initiation of hemodialysis was the highest in winter and the lowest in summer [12].

Similar results were obtained in the present study. The number of deaths due to renal failure was the highest in January in Hokkaido, the northernmost point of Japan. However, the relationship between the numbers of deaths due to renal failure and air temperature parameters in Okinawa remains unclear. Decreases in water intake due to lower air temperatures [20], higher blood pressure [21], and lower physical activity [22] have been reported in winter. These factors may influence the relationship between the number of deaths due to renal failure and air temperature parameters in Hokkaido, Japan. Nevertheless, the present results suggest that a larger number of deaths due to renal failure may be more common in an area with a low air temperature. Therefore, further studies are needed on changes in renal function in clinical settings in winter using blood sampling data.

There are some limitations that need to be addressed. The present study was an ecological study and, thus, the results obtained may not apply to individuals. Furthermore, individual data were not evaluated. Moreover, the relationship between the number of deaths due to renal failure and air temperature parameters has not yet been elucidated in detail. In conclusion, lower air temperature was closely associated with the number of deaths due to renal failure, particularly in Hokkaido, Japan. Further studies using individual data are warranted.

## 5. Conclusions

The present results suggest that the relationship between the number of deaths due to renal failure and air temperature parameters differs between Hokkaido and Okinawa. Lower air temperature was thought to be closely associated with the number of deaths due to renal failure, particularly in Hokkaido, Japan. Further studies using individual data would be needed in the future.

## Figures and Tables

**Table 1:** Clinical data on the number of deaths due to renal failure and air temperature parameters in Hokkaido and Okinawa, Japan.

	Hokkaido			Okinawa			*p*
	Mean ± SD	Minimum	Maximum		Minimum	Maximum	
Number of months	108			108			
Number of deaths due to renal failure (total)	2.28 ± 0.30	1.66	3.04	1.17 ± 0.31	0.50	2.00	<0.01
Number of deaths due to renal failure (men)	1.09 ± 0.19	0.78	1.66	0.51 ± 0.20	0.14	1.04	<0.01
Number of deaths due to renal failure (women)	1.19 ± 0.18	0.81	1.70	0.71 ± 0.24	0.27	1.73	<0.01
Mean air temperature (°C)	9.47 ± 9.27	−4.70	24.80	23.31 ± 4.49	14.90	29.80	<0.01
Mean of the highest air temperature (°C)	13.37 ± 9.86	−2.00	29.10	26.02 ± 4.57	17.00	32.90	<0.01
Mean of the lowest air temperature (°C)	6.00 ± 9.19	−8.00	21.30	21.11 ± 4.62	12.70	27.60	<0.01
The highest air temperature (°C)	20.60 ± 9.76	1.80	34.50	29.36 ± 3.44	22.60	34.80	<0.01
The lowest air temperature (°C)	0.77 ± 9.40	−14.30	16.90	17.45 ± 5.43	6.10	25.70	<0.01

Number of deaths due to renal failure: per 100,000 people/month.

**Table 2:** Relationship between the number of deaths due to renal failure and air temperature parameters.

	Hokkaido						Okinawa					
	Total		Men		Women		Total		Men		Women	
	*r*	*p*	*r*	*p*	*r*	*p*	*r*	*p*	*r*	*p*	*r*	*p*
Mean air temperature (°C)	−0.51	**<0.01**	−0.39	**<0.01**	−0.44	**<0.01**	−0.20	**0.04**	−0.20	**0.04**	−0.17	0.08
Mean of the highest air temperature (°C)	−0.51	**<0.01**	−0.39	**<0.01**	−0.44	**<0.01**	−0.19	**0.04**	−0.20	**0.04**	−0.16	0.10
Mean of the lowest air temperature (°C)	−0.50	**<0.01**	−0.39	**<0.01**	−0.43	**<0.01**	−0.21	**0.03**	−0.21	**0.03**	−0.17	0.08
The highest air temperature (°C)	−0.52	**<0.01**	−0.41	**<0.01**	−0.44	**<0.01**	−0.22	**0.02**	−0.21	**0.03**	−0.16	0.09
The lowest air temperature (°C)	−0.49	**<0.01**	−0.36	**<0.01**	−0.44	**<0.01**	−0.19	0.05	−0.18	0.06	−0.17	0.09

**Table 3:** Comparison of the number of deaths due to renal failure classified by month in Hokkaido and Okinawa, Japan.

	Hokkaido						Okinawa		
	Total		Men		Women		Total	Men	Women
January	2.67 ± 0.29		1.31 ± 0.24		1.36 ± 0.14		1.32 ± 0.36	0.59 ± 0.21	0.77 ± 0.23
February	2.26 ± 0.25		1.05 ± 0.13		1.21 ± 0.19		1.25 ± 0.42	0.58 ± 0.26	0.73 ± 0.26
March	2.34 ± 0.31		1.12 ± 0.19		1.22 ± 0.18		1.17 ± 0.29	0.50 ± 0.18	0.76 ± 0.41
April	2.22 ± 0.31		1.00 ± 0.14		1.21 ± 0.25		1.24 ± 0.34	0.54 ± 0.22	0.74 ± 0.19
May	2.31 ± 0.25		1.11 ± 0.18		1.20 ± 0.13		1.15 ± 0.31	0.54 ± 0.16	0.68 ± 0.33
June	2.01 ± 0.19	a	0.96 ± 0.12	a	1.05 ± 0.14	a	1.01 ± 0.39	0.35 ± 0.23	0.69 ± 0.20
July	2.06 ± 0.22	a	1.03 ± 0.16		1.04 ± 0.09	a	1.06 ± 0.21	0.51 ± 0.17	0.58 ± 0.17
August	2.05 ± 0.20	a	0.97 ± 0.18		1.08 ± 0.10	a	1.23 ± 0.28	0.53 ± 0.16	0.76 ± 0.21
September	2.18 ± 0.13	a	0.97 ± 0.12	a	1.22 ± 0.17		1.07 ± 0.34	0.48 ± 0.26	0.62 ± 0.14
November	2.45 ± 0.27		1.18 ± 0.21		1.27 ± 0.12		1.10 ± 0.26	0.47 ± 0.13	0.70 ± 0.15
December	2.49 ± 0.24		1.19 ± 0.14		1.30 ± 0.14		1.23 ± 0.23	0.57 ± 0.18	0.77 ± 0.28

a: *p* < 0.05 vs. January; per 100,000 people/month. Comparisons of the number of deaths due to renal failure between January and other months were conducted using the Steel test.
